# Fracking compared to conventional balloon angioplasty alone for calcified common femoral artery lesions using intravascular ultrasound analysis: 12-month results

**DOI:** 10.1186/s42155-023-00373-y

**Published:** 2023-04-20

**Authors:** Takuya Haraguchi, Tsutomu Fujita, Yoshifumi Kashima, Masanaga Tsujimoto, Ryo Otake, Yuhei Kasai, Katsuhiko Sato

**Affiliations:** Department of Cardiology, Asia Medical Group, Sapporo Heart Center, Sapporo Cardio Vascular Clinic, North 49, East 16, 8-1, Higashi Ward, Sapporo City, Hokkaido 007-0849 Japan

**Keywords:** Common femoral artery, Calcification, Fracking, Atherectomy, Lithoplasty, Peripheral artery disease, Endovascular treatment, Balloon angioplasty

## Abstract

**Background:**

Fracking is a novel technique to crack calcified lesions by hydraulic pressure. This study aimed to compare the performance of fracking and conventional balloon angioplasty without stenting for calcified common femoral artery (CFA) lesions using intravascular ultrasound (IVUS) analysis.

**Methods:**

This retrospective, single-center, comparative observational study included 59 patients (67 limbs) with calcified CFA lesions treated with either fracking (*n* = 30) or balloon angioplasty (*n* = 29) between January 2018 and December 2020. The primary endpoint was 1-year primary patency. The secondary endpoints included procedure success, freedom from target lesion revascularization (TLR), procedure-related complications, and freedom from major adverse limb events (MALE). Predictors of restenosis were identified using multivariate Cox proportional hazards analysis.

**Results:**

The mean follow-up duration was 403 ± 236 days. The fracking group had significantly higher incidence of 1-year primary patency (89.8% versus 49.2%, *P <* 0.001), procedure success (96.9% versus 74.3%, *P =* 0.009), and freedom from TLR (93.5% versus 74.2%, *P =* 0.038) than the balloon group. The rate of freedom from MALE was significantly higher in the fracking group than in the balloon group (76.9% versus 48.6%, *P =* 0.033). The groups had no significant difference in procedure-related complications (6.2% versus 5.7%, *P =* 0.928). A larger postprocedural IVUS-estimated minimum lumen area (MLA) was associated with a lower risk of restenosis (hazard ratio, 0.78; 95% confidence interval, 0.67–0.91; *P <* 0.001), with a cut-off value of 16.0 mm^2^ determined using receiver operating characteristics curve analysis. The incidence of 1-year primary patency in patients with a postprocedural MLA ≥16.0 mm^2^ (*n* = 37) was significantly higher than that in those with a postprocedural MLA < 16.0 mm^2^ (*n* = 30) (87.8% versus 44.6%, *P <* 0.001).

**Conclusion:**

This study demonstrated the superior procedural efficacy of fracking compared to balloon angioplasty in treating calcified CFA lesions. The safety outcomes after fracking were comparable to those after balloon angioplasty. Large postprocedural MLA was an independent positive predictor of patency.

## Background

Lower extremity artery disease often involves the common femoral artery (CFA), which is the main arterial supply to the lower extremities (Mukherjee and Inahara 1989; Fowkes et al. 2013). Surgical endarterectomy is considered the standard treatment for symptomatic CFA diseases (Aboyans et al. 2018; Gerhard-Herman et al. 2017); however, it has a perioperative complication rate ranging from 6 to 17%. Peripheral interventions, such as conventional balloon and drug-coated balloon (DCB) angioplasty, stent implantation, atherectomy, and lithoplasty have been established as less invasive options and have demonstrated efficacy against restenosis for treating CFA diseases in recent years (Siracuse et al. 2014; de Blic et al. 2015; Thiney et al. 2015; Gallagher et al. 2011; Gouëffic et al. 2017; Brodmann et al. 2019; Kuo et al. 2019). However, evidence supporting the equivalence of peripheral intervention to surgical endarterectomy is insufficient.

Fracking is a novel technique that utilizes hydraulic pressure to fracture deep calcifications through a needle puncture in calcified plaque (Haraguchi et al. 2021). Fracking has been shown to achieve a more significant acute luminal gain than both balloon angioplasty and percutaneous direct needle puncture of calcified plaque (Haraguchi et al. 2021; Ichihashi et al. 2014). However, the long-term clinical benefits of fracking in the treatment of CFA disease have yet to be established. This study aimed to compare the safety and efficacy of fracking versus conventional balloon angioplasty for the treatment of calcified CFA lesions up to 1 year.

## Methods

### Study design and patient population

This retrospective, single-center, observational study was conducted at the Cardiology Department of Sapporo Heart Center in Japan. Between January 2018 and December 2020, 138 patients underwent endovascular interventions for CFA disease. Eligible patients had calcified CFA lesions with Rutherford classification 2 to 6 and received treatment using intravascular ultrasound (IVUS) without stent implantation. Of these, 79 patients were excluded from the study due to acute limb ischemia (8), in-stent lesions (7), absence of calcified lesions (31), non-use of IVUS (19), or insufficient data for analysis (14).

### Procedures and IVUS analysis

The procedures involved administering local anesthesia with 2.0% xylocaine, followed by performing a crossover approach to insert a 6-Fr sheath through the CFA opposite the calcified lesions in eligible patients. A 5000-IU dose of unfractionated heparin was injected intra-arterially with a goal-activated coagulation time > 250 seconds. After crossing the lesion with a 0.014- or 0.018-in. hydrophilic guidewire, IVUS was performed before and after revascularization using a 20-MHz Vision PV18 (Philips Volcano, San Diego, California) or 30-MHz OptiCross18 (Boston Scientific Co., Marlborough, Massachusetts). A balloon with a diameter determined by IVUS measurement estimates was used to ensure maximum lumen area. After balloon angioplasty, lesions with residual angiographic stenosis of > 50% or inadequate lumen area by IVUS estimation were additionally treated with the fracking technique. Scoring balloons (AngioSculpt: Philips, San Diego, California, USA and Cutting balloon: Boston Scientific, Marlborough, Massachusetts, USA) and DCB (IN.PACT Admiral, Medtronic, Santa Clara, USA) were predominantly used for calcifications within the 180–360° range, as evaluated by IVUS. Final angiography and IVUS assessments were conducted in all cases. Upon successful completion of three fracking cases, the operator demonstrated the ability to perform the procedure independently. Consequently, one skilled operator and two general operators, each with experience in completing a minimum of three fracking cases, conducted the procedure in this study.

**Fig. 1:**
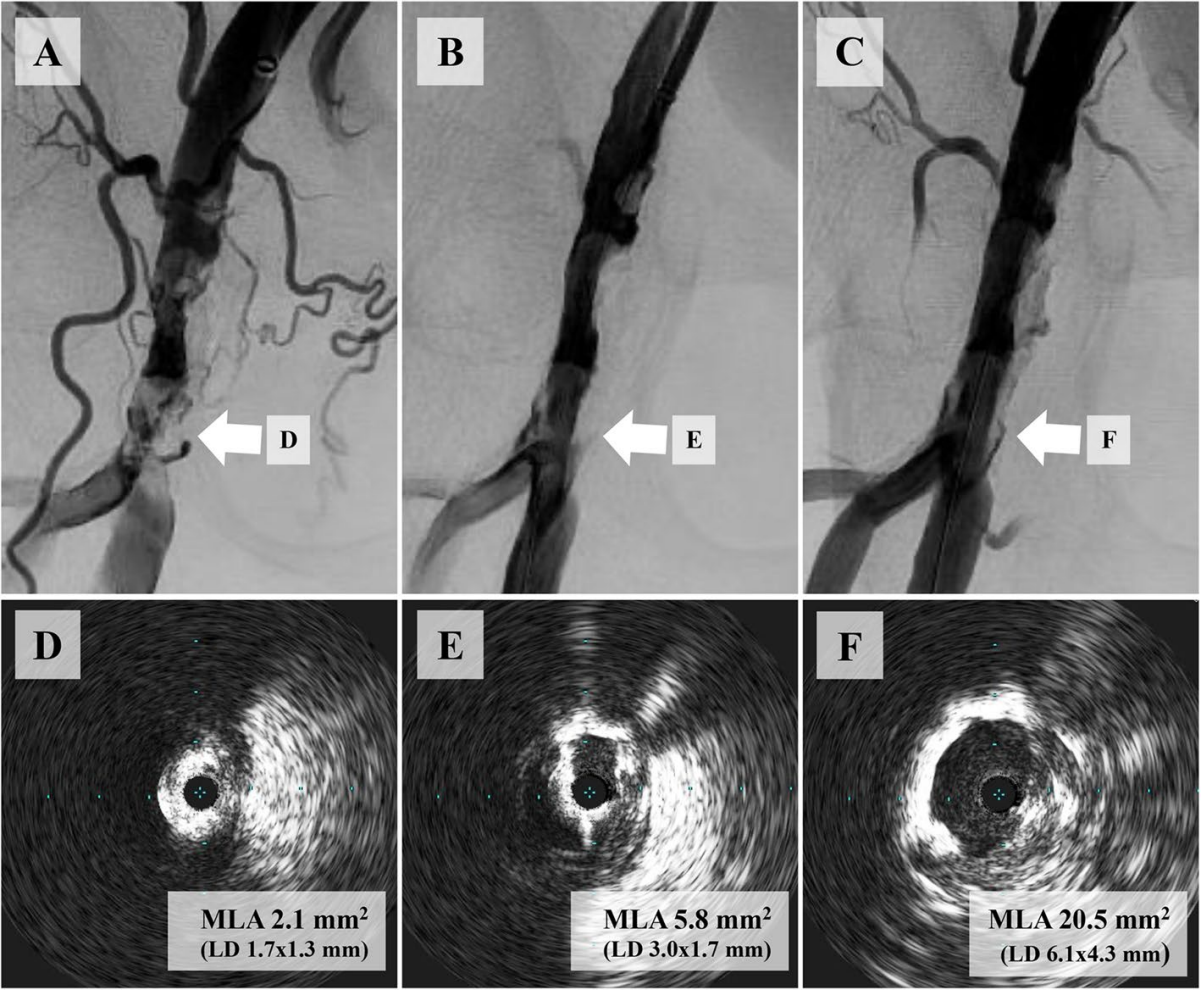
Representative case of lesion morphology and IVUS-evaluated MLA before and after fracking and balloon angioplasty. **A** Initial angiography shows a severely calcified plaque in the right CFA. **B** Initial IVUS evaluation demonstrates a preprocedural MLA of 2.1 mm^2^ (LD, 1.7 × 1.3 mm). **C**, **D** Subsequent angiography and IVUS show residual stenosis of 33% and a periprocedural MLA of 5.8 mm^2^ (LD, 3.0 × 1.7 mm) after balloon angioplasty using a 7.0 mm noncompliant balloon. **E** After fracking, the final angiography shows a satisfactory image with a stenosis rate of 17%. **F** Post-fracking IVUS evaluation demonstrates a postprocedural MLA of 20.5 mm^2^ (LD, 6.1 × 4.3 mm). Abbreviations: IVUS, intravascular ultrasound; LD, lumen diameter; MLA, minimum lumen area

In fracking, an 18-gauge needle (Terumo Corporation, Tokyo, Japan) without a plastic outer sheath was inserted into the area of calcification that showed inadequate expansion on angiography or IVUS (Haraguchi et al. 2021). Balloon dilation was maintained during the puncture to compress the calcification, increase its density, and prevent dissection which might spread extensively due to the hydraulic pressure applied during the fracking procedure. After needle insertion, a 3.0-ml lock syringe was attached to the needle to confirm the location of the needle tip inside the plaque by injecting saline from the syringe. The “fracking point” location was determined when the needle tip reached dense areas of calcification and the syringe plunger could not be pushed further manually. A balloon indeflator with a half concentration of contrast agent was connected to the needle, and the pressure of the indeflator was gradually increased and applied to the densely calcified plaque under angiography until a sudden reduction in pressure was observed, indicating successful cracking of the calcification. This process is known as “fracking.” Fracking was repeatedly performed at several fracking points until the operator could no longer detect fracking points or a sufficient minimum lumen area (MLA) determined by IVUS was obtained. Dilatation using the previous balloon was performed to compress the calcification further. The procedure was considered complete when IVUS demonstrated that the targeted MLA or more had been achieved. A representative case is shown in Fig. 1.

In IVUS analysis, proximal and distal reference segments were defined as the most normal cross-sections in the same arterial segment within 20 mm proximal and distal to the target lesion, respectively, but before any large side branch (Miki et al. 2016). The lumen and external elastic membrane cross-sectional area were measured at the proximal and distal reference segments, and the image slice with the MLA was selected for analysis before and after lesion dilatation. The calcification in lesions was categorized according to the Peripheral Arterial Calcium-Scoring System (PACSS) under angiography (Rocha-Singh et al. 2014). The circumferential grade of calcified plaque was determined using IVUS based on the presence of calcium plaques in the 1–180° and 180–360° ranges of calcification (Fujihara et al. 2019).

All patients received dual antiplatelet drugs after the procedure for at least 8 weeks, followed by monotherapy. Patients who were previously on anticoagulant therapy were additionally administered an antiplatelet agent after the procedure for 8 weeks, followed by anticoagulant monotherapy.

### Endpoints

The primary endpoint was 1-year primary patency which was defined as patency of a target lesion without significant stenosis (> 2.4 on duplex ultrasonography or > 50% stenosis on digital subtraction or computed tomography angiography) without target lesion revascularization (TLR). The secondary endpoints included procedure success, defined as < 30% residual stenosis without suboptimal results; freedom from TLR, defined as the absence of repeat endovascular or surgical bypass procedures for limbs with recurrent symptoms accompanied by recurrent stenosis > 50% as measured by angiography; procedure-related complications, defined as any complications related to the intervention; and freedom from major adverse limbs event (MALE), defined as any reintervention, major amputation, or all-cause death.

Patients were followed up regularly for 1, 6, and 12 months or in cases of clinical worsening at our outpatient department. The follow-up protocol included the postoperative ankle-brachial index (ABI) and lower limb duplex ultrasound at every follow-up. If the ultrasound scan was inconclusive, a computed tomography angiogram, digital subtraction angiography, or both were performed, following a prespecified protocol to determine if the endpoints were achieved.

### Statistical analysis

Continuous variables are presented as the mean ± standard deviation, while categorical variables are reported as counts and percentages. The chi-square test or two-sided Fisher’s exact test was used to compare statistical significance between the two groups for categorical variables. Two-sided t-tests were used to obtain *P*-values for continuous variables. Time-to-event outcomes were analyzed with Kaplan–Meier curves for primary patency and TLR up to 400 days. Patency loss was primarily compared between groups with the log-rank test. A *P*-value < 0.05 was considered statistically significant. Independent outcome determinants of primary patency were determined by the Cox proportional hazards regression model with multivariate analysis, including all univariable parameters with a *P*-value < 0.10. Receiver operating characteristic (ROC) curve analysis was performed to assess the ability of postprocedural MLA to resist restenosis. The area under the ROC curve was calculated for postprocedural MLA. All analyses were performed using SPSS statistics version 29.0 (SPSS, Inc., Chicago, IL, USA).

## Results

### Baseline characteristics

During the study period, 59 patients were treated for a total of 67 atherosclerotic CFA lesions, with 30 patients (32 lesions) in the fracking group and 29 patients (35 lesions) in the balloon group. Baseline patient characteristics, as presented in Table 1, were generally well balanced between the groups, except for a higher proportion of male patients in the fracking group (76.7% versus 51.7%, *P =* 0.046). Lesion and procedure characteristics are summarized in Table 2. CFA lesions involving proximal superficial femoral artery disease were more frequently observed in the balloon group than in the fracking group (68.6% versus 31.3%, *P =* 0,002). The fracking group had higher rates of preoperative stenosis (96.5 ± 6.4% versus 87.9 ± 10.2%, *P <* 0.001), bilateral calcification (81.3% versus 34.3%, *P <* 0.001), and severe PACSS-graded calcification (3.0 ± 1.0 versus 1.9 ± 1.0, *P <* 0.001) compared to the balloon group. The proportion of DCB use was comparable in both groups (34.3% versus 34.3%, *P =* 0.994). Radiation time in the fracking group was significantly shorter than that in the balloon group (25.5 ± 13.8 min versus 51.6 ± 44.3 min, *P =* 0.002). The average number of fracking procedures per case was 2.4 ± 0.8. The average procedure time for fracking was 10.1 ± 3.8 minutes. The residual stenosis in the fracking group was much smaller than that in the balloon group (19.5 ± 12.3% versus 28.6 ± 19.3%, *P =* 0.027). The postoperative ABI improved more significantly in the fracking group than in the balloon group (0.99 ± 0.20 versus 0.78 ± 0.28, *P <* 0.001).

**Table 1:** Patient characteristics

Variables	Total *n* = 59	Fracking *n* = 30	Balloon *n* = 29	*P*-value
Age, y	77.0 ± 8.0	77.3 ± 8.4	76.7 ± 7.8	0.774
Men	38 (64)	23 (77)	15 (52)	0.046
Body mass index, kg/m^2^	22.0 ± 3.7	21.3 ± 2.9	22.8 ± 4.2	0.130
Current smoker	12 (20)	6 (20)	6 (21)	0.949
Hypertension	52 (88)	25 (83)	27 (93)	0.253
Dyslipidemia	43 (73)	21 (70)	22 (76)	0.620
Diabetes mellitus	39 (66)	17 (57)	22 (76)	0.124
Chronic kidney disease	40 (68)	20 (67)	20 (69)	0.853
Hemodialysis	18 (30)	10 (33)	8 (28)	0.639
Heart failure	11 (19)	5 (17)	6 (21)	0.698
Coronary artery disease	44 (75)	20 (67)	24 (83)	0.161
Cerebrovascular disease	6 (10)	4 (13)	2 (7)	0.422
Aspirin	54 (91)	26 (87)	28 (96)	0.179
P2Y12 Inhibitor	44 (75)	22 (73)	22 (76)	0.827
Cilostazol	17 (29)	9 (30)	8 (28)	0.841
Anti-coagulant	8 (14)	4 (13)	4 (14)	0.960
Statin	30 (51)	18 (60)	12 (41)	0.158

Continuous variables are indicated as mean ± standard deviation. Categorical variables are expressed as the counts (percentage)

**Table 2:** Lesion and procedure characteristics

Variables	Total *n* = 67	Fracking *n* = 32	Balloon *n* = 35	*P*-value
Rutherford classification	3.3 ± 0.9	3.1 ± 0.8	3.4 ± 1.0	0.143
Chronic limb-threatening ischemia	16 (24)	5 (16)	9 (31)	0.134
Preoperative ankle-brachial index	0.45 ± 0.32	0.52 ± 0.29	0.39 ± 0.34	0.095
De novo lesion	59 (88)	29 (91)	30 (86)	0.543
Lesions only in CFA region	33 (49)	22 (69)	11 (31)	0.002
Deep femoral artery disease	17 (26)	7 (22)	10 (29)	0.536
Focal lesion	27 (40)	13 (41)	14 (40)	0.959
Preoperative stenosis (%)	92.0 ± 9.6	96.5 ± 6.4	87.9 ± 10.2	< 0.001
Proximal reference vessel diameter (mm)	6.3 ± 0.8	6.4 ± 0.9	6.2 ± 0.8	0.344
Distal reference vessel diameter (mm)	5.6 ± 0.8	5.8 ± 0.9	5.5 ± 0.7	0.165
Lesion length (mm)	38.7 ± 14.3	38.4 ± 14.6	38.8 ± 14.3	0.906
Chronic total occlusion	11 (16)	4 (12)	7 (20)	0.415
length (mm)	30.0 ± 8.9	27.5 ± 12.6	31.4 ± 6.9	0.512
PACSS grade	2.4 ± 1.1	3.0 ± 1.0	1.94 ± 1.0	< 0.001
1, 2	21 (31), 8 (12)	5 (16), 1 (3)	16 (46), 7 (20)	0.007, 0.034
3, 4	25 (37), 13 (19)	15 (47), 11 (34)	10 (29), 2 (6)	0.125, 0.003
Bilateral calcium	38 (57)	26 (81)	12 (34)	< 0.001
Below-the-knee arterial run-off	1.7 ± 0.9	1.9 ± 0.9	1.5 ± 0.9	0.086
Noncompliant balloon use	39 (58)	25 (78)	27 (77)	0.925
Scoring and cutting balloon use	45 (67)	22 (69)	23 (66)	0.795
Drug-coated balloon use	23 (34)	11 (34)	12 (34)	0.994
Postprocedural dissection, none to mild	50 (75)	21 (66)	29 (83)	0.109
Procedure time (min)	66.9 ± 50.7	56.0 ± 24.0	76.9 ± 65.2	0.092
Contrast dose (ml)	132.9 ± 55.7	121.4 ± 36.3	143.4 ± 67.7	0.107
Radiation time (min)	39.2 ± 35.7	25.5 ± 13.8	51.6 ± 44.3	0.002
Number of Fracking	–	2.4 ± 0.8	–	–
Fracking procedure time (min)	–	10.1 ± 3.8	–	–
Postoperative stenosis (%)	24.2 ± 16.8	19.5 ± 12.3	28.6 ± 19.3	0.027
Postoperative ankle-brachial index	0.88 ± 0.27	0.99 ± 0.20	0.78 ± 0.28	< 0.001
IVUS examination				
Proximal reference vessel area (mm^2^)	55.0 ± 15.1	54.8 ± 14.8	55.1 ± 15.6	0.947
Lesion vessel area (mm^2^)	47.6 ± 11.6	46.0 ± 11.5	49.0 ± 11.6	0.302
Preoperative MLA (mm^2^)	5.8 ± 3.4	5.7 ± 3.1	5.9 ± 3.7	0.839
Preoperative plaque burden (%)	87 ± 8	86 ± 8	87 ± 8	0.706
Distal reference vessel area (mm^2^)	40.2 ± 14.4	37.3 ± 15.1	42.9 ± 13.3	0.110
Calcium angle ≥180–360°	43 (74)	26 (81)	17 (49)	0.005
Postoperative MLA (mm^2^)	17.2 ± 6.0	22.1 ± 4.0	12.7 ± 3.3	< 0.001
Acute gain (mm^2^)	11.2 ± 6.8	16.4 ± 4.8	6.8 ± 4.4	< 0.001
Postoperative plaque burden (%)	61 ± 17	49 ± 16	73 ± 9	< 0.001

Continuous variables are indicated as mean ± standard deviation. Categorical variables are expressed as the counts (percentage)

*Abbreviations*: *CFA* common femoral artery, *IVUS* intravascular ultrasound, *MLA* minimum lumen area, *PACSS* Peripheral Artery Calcium Scoring System

The IVUS examination, as shown in Table 2, revealed no significant differences in the fundamental examination values between the groups. However, more severe calcification of 180–360° was noted in the fracking group than in the balloon group (81.3% versus 48.6%, *P =* 0.005). Although the MLAs before dilatation were similar in both groups (5.7 ± 3.1 mm^2^ versus 5.9 ± 3.7 mm^2^, *P =* 0.839), the MLAs after dilatation (22.1 ± 4.0 mm^2^ versus 12.7 ± 3.3 mm^2^, *P <* 0.001) and acute luminal gain (16.4 ± 4.8 mm^2^ versus 6.8 ± 4.4 mm^2^, *P <* 0.001) were more prominent in the fracking group than in the balloon group. Additionally, the preoperative plaque burden (86 ± 8% versus 87 ± 8%, *P =* 0.706) was not significantly different between the groups, but the postoperative plaque burden was lower in the fracking group than in the balloon group (49 ± 16% versus 73 ± 9%, *P <* 0.001).

### Follow-up outcomes and predictors of restenosis

**Fig. 2:**
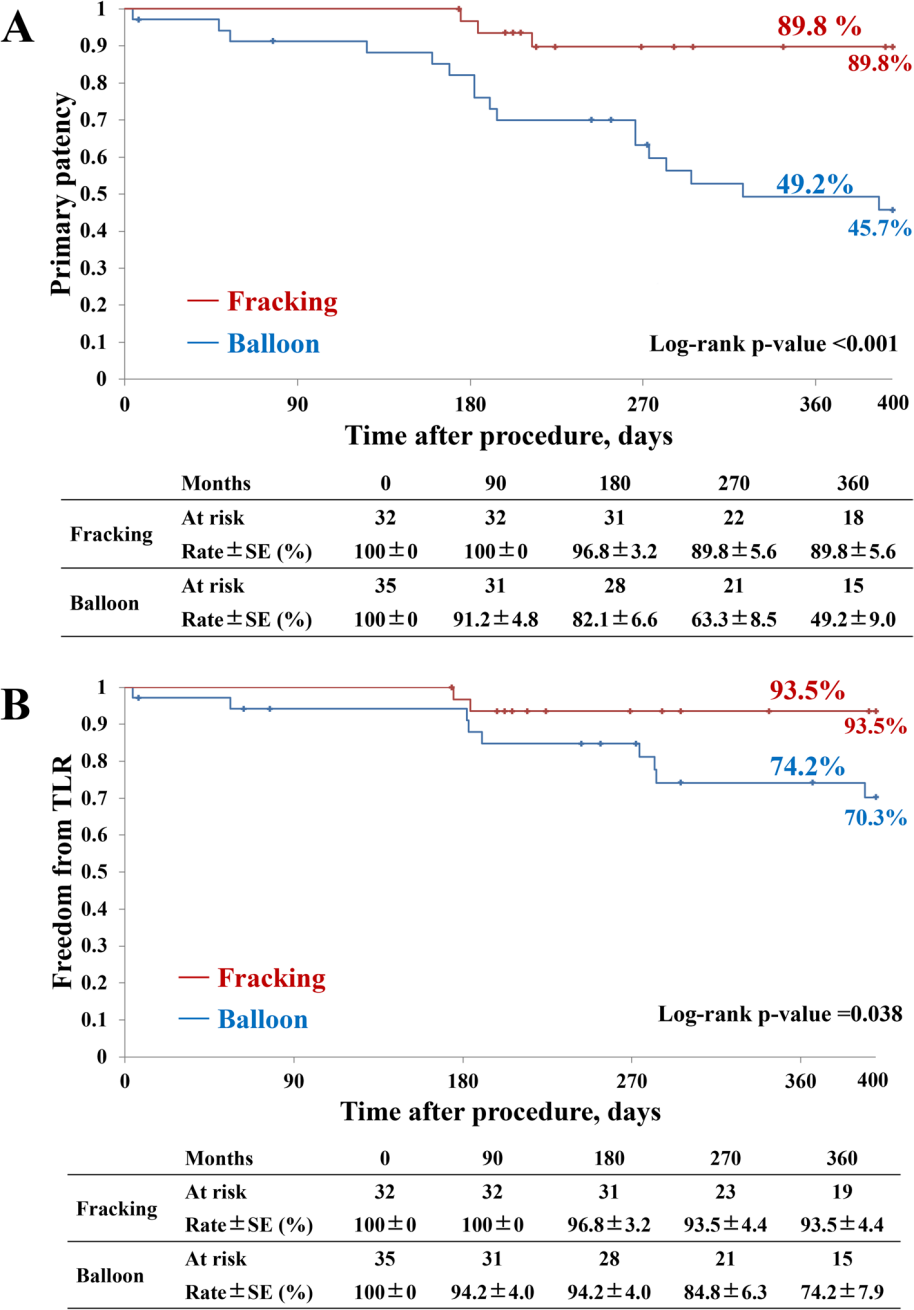
Comparison of primary patency and freedom from TLR between fracking and conventional balloon angioplasty. **A** Primary patency and (**B**) freedom from target lesion revascularization rate for up to 1 year and 1 month after the fracking and balloon angioplasty procedures are shown. The figures show that both primary patency and freedom from TLR rates were significantly higher in the fracking group than in the balloon group

**Fig. 3:**
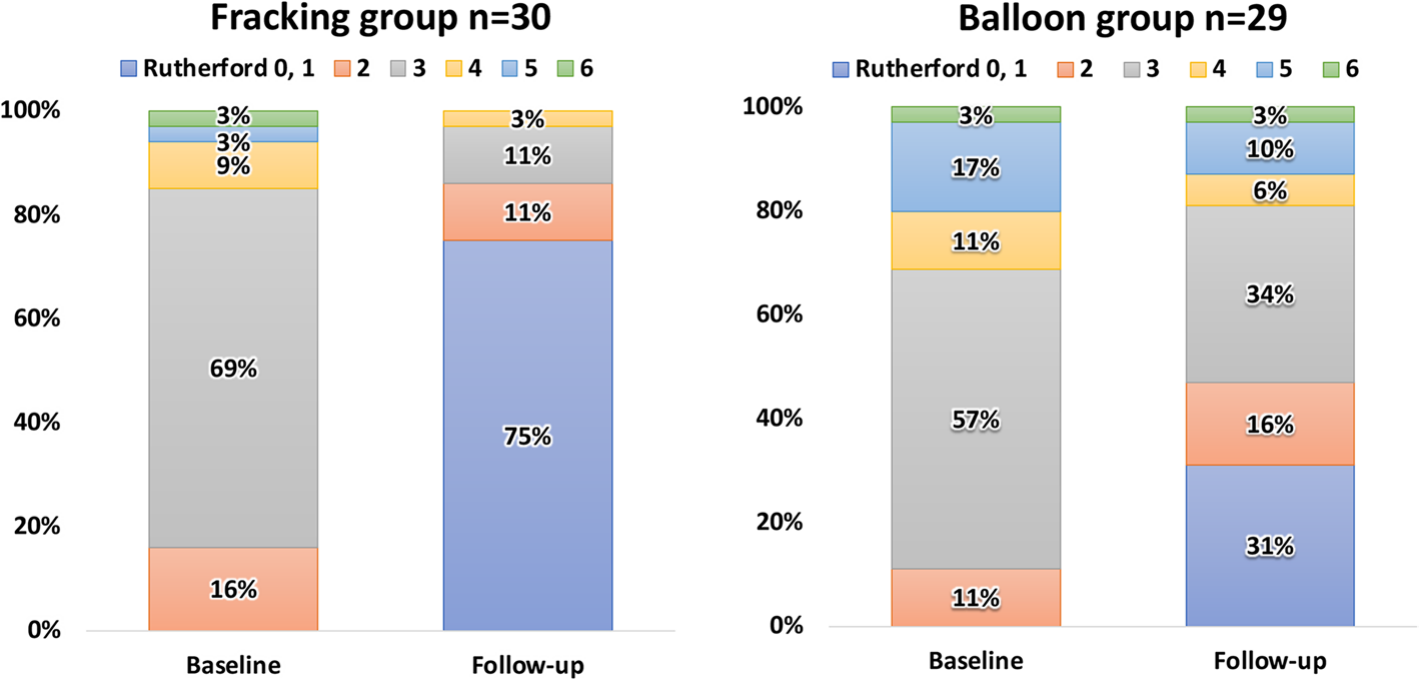
Change in Rutherford classification for the fracking and the balloon groups at baseline and follow-up. The Rutherford classification improved similarly in both groups

During the 1-year follow-up (mean 403.2 ± 236.0 days), Table 3 demonstrates that the fracking group had a significantly higher 1-year primary patency rate compared to the balloon group (89.8% versus 49.2%, *P <* 0.001), as shown in Fig. 2A. The procedure success rate was also significantly higher in the fracking group than in the balloon group (96.9% versus 74.3%, *P =* 0.009). Furthermore, the fracking group had a significantly higher incidence of freedom from TLR than the balloon group (93% versus 74%, *P =* 0.038), as shown in Fig. 2B. The incidence of procedure-related complications was comparable between the two groups (6.2% versus 5.7%, *P =* 0.928). No complications at the target lesions were documented. Hemorrhage at the sheath insertion site was noted in two cases in the fracking group. The fracking group had a significantly higher rate of freedom from MALE than the balloon group (76.9% versus 48.6%, *P =* 0.033). Similarly, the Rutherford classification improved in both groups, as shown in Fig. 3.

**Table 3:** Perioperative and 1-year clinical outcomes

Variables	Total *n* = 67	Fracking *n* = 32	Balloon *n* = 35	*P*-value
Mean follow-up duration (days)	403 ± 236	432 ± 222	377 ± 248	0.339
Procedure success	57 (85.1)	31 (96.9)	26 (74.3)	0.009
Primary patency^a^	48 (67.9)	29 (89.8)	19 (49.2)	<.001
Restenosis	18 (26.9)	3 (9.4)	15 (42.8)	0.002
Reocclusion	2 (3.0)	0 (0)	2 (5.7)	0.269
Freedom from TLR^a^	57 (83.1)	30 (93.5)	27 (74.2)	0.038
Freedom from MALE	29 (61.6)	16 (76.9)	13 (48.6)	0.033
Freedom from any reintervention	32 (70.5)	16 (85.7%)	16 (57.2)	0.015
All-cause death^a^	7 (10.0)	4 (13.6)	3 (11.5)	0.719
Major amputation^a^	2 (3.1)	1 (3.2)	1 (2.9)	0.974
Procedure-related complications	4 (6.0)	2 (6.2)	2 (5.7)	0.928

Continuous variables are indicated as mean ± standard deviation. Categorical variables are expressed as the counts (percentage)

^a^The results were analyzed with Kaplan-Meier curves

*Abbreviations*: *MALE* major adverse limbs event, *TLR* target lesion revascularization

**Fig. 4:**
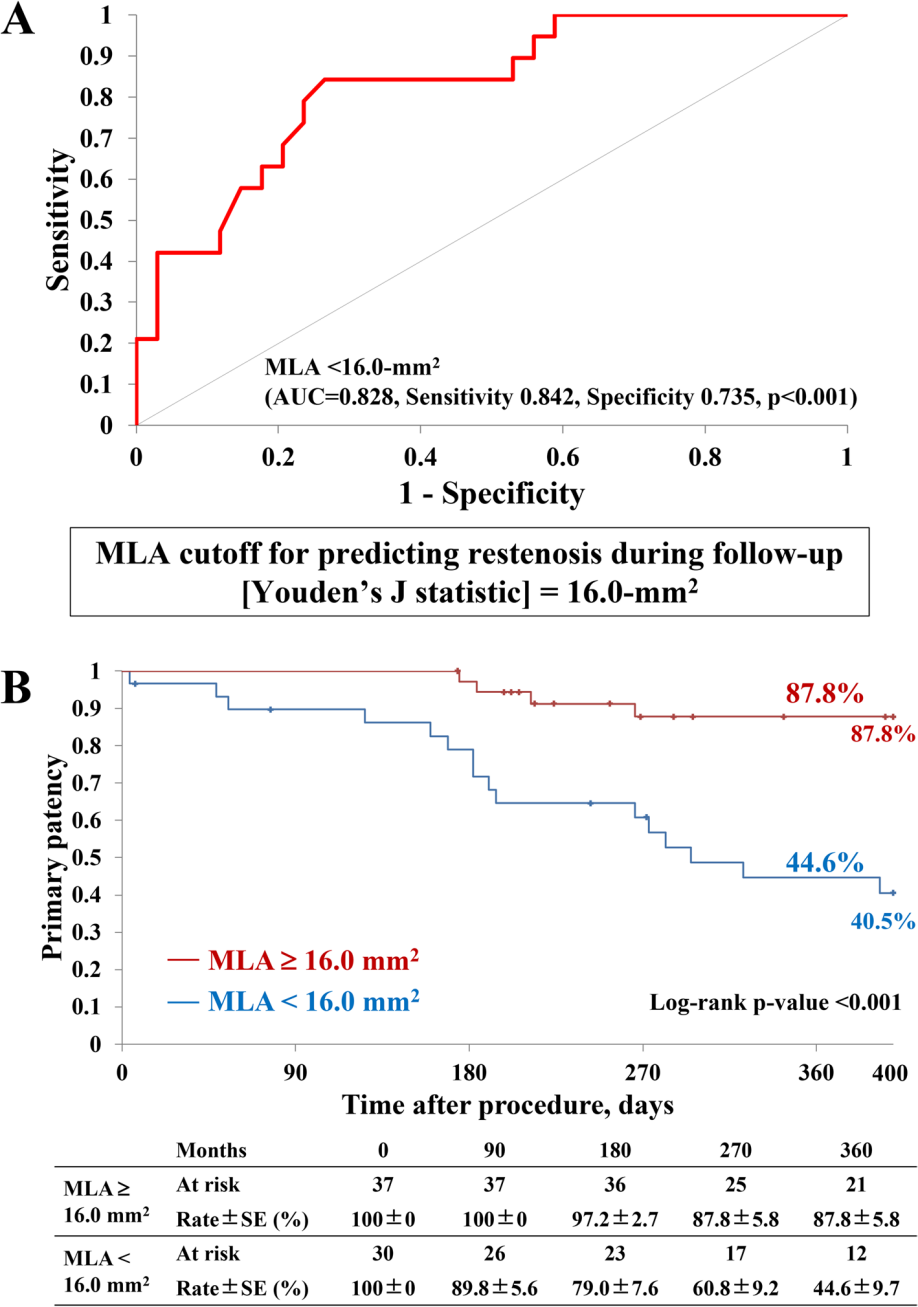
Comparison of primary patency between optimal (≥16.0 mm^2^) and suboptimal (< 16.0 mm^2^) postprocedural MLA. **A** The ROC curve analysis generated a cut-off value of 16.0 mm^2^ of postprocedural MLA to predict 1-year restenosis. **B** The incidence of 1-year primary patency was significantly higher in patients with an optimal postprocedural MLA (≥16.0 mm^2^) than in those with a suboptimal postprocedural MLA (< 16.0 mm^2^). Abbreviations: MLA, minimum lumen area; ROC, receiver operating characteristic

Multivariate Cox proportional hazards analysis, as presented in Table 4, showed that a large postprocedural MLA (per 1.0 mm^2^) was an independent predictor of protection against restenosis (hazard ratio, 0.78; 95% confidence interval, 0.67–0.91; *P <* 0.001). Furthermore, the ROC curve analysis in Fig. 4A indicated a cut-off value of 16.0 mm^2^ of postprocedural MLA that prevented 1-year restenosis after CFA treatment, with an area under the curve of 0.828 (sensitivity 0.842, specificity 0.735, *P <* 0.001). The incidence of 1-year primary patency was significantly higher in patients (*n* = 37) with a postprocedural MLA ≥16.0 mm^2^ than in those (*n* = 30) with a postprocedural MLA < 16.0 mm^2^ (87.8% versus 44.6%, *P <* 0.001), as shown in Fig. 4B.

**Table 4:** Interaction effect on the association of fracking versus conventional balloon angioplasty with 1-year restenosis

	Univariate analysis		Multivariate analysis	
	HR (95% CI)	*P*-value	HR (95% CI)	*P*-value
Age ≥ 80, y	0.37 (0.13–1.03)	0.056	0.39 (0.14–1.08)	0.071
Diabetes mellitus	2.03 (0.68–6.07)	0.207		
Chronic kidney disease	0.82 (0.33–2.01)	0.666		
Hemodialysis	1.59 (0.63–4.01)	0.330		
Chronic limb-threatening ischemia	1.31 (0.47–3.60)	0.604		
Lesions only in CFA region	0.72 (0.30–1.74)	0.467		
No deep femoral artery lesions	0.51 (0.21–1.24)	0.138		
Chronic total occlusion	1.64 (0.60–4.52)	0.337		
Focal lesion	1.30 (0.54–3.15)	0.555		
De novo lesion	1.08 (0.25–4.66)	0.919		
Distal reference vessel diameter (per 1.0 mm)	0.90 (0.52–1.54)	0.693		
Lesion length (per 1.0 mm)	0.99 (0.97–1.03)	0.919		
PACSS grade	0.88 (0.60–1.30)	0.528		
Bilateral calcified lesions	0.77 (0.32–1.84)	0.551		
No below-the-knee artery run-off	0.91 (0.21–3.94)	0.903		
Noncompliant balloon use	1.68 (0.49–5.74)	0.407		
Scoring/Cutting balloon use	0.90 (0.36–2.25)	0.815		
Drug coating balloon use	0.74 (0.28–1.93)	0.538		
Residual stenosis	1.01 (0.99–1.04)	0.229		
Dissection, none to mild	1.14 (0.38–3.41)	0.819		
Calcium angle ≥180–360° by IVUS analysis	1.21 (0.46–3.15)	0.694		
Postoperative MLA (per 1.0 mm^2^)	0.85 (0.78–0.93)	< 0.001	0.78 (0.67–0.91)	< 0.001
Acute gain (per 1.0 mm^2^)	0.93 (0.86–0.99)	0.038	1.10 (0.96–1.27)	0.162

Data are presented as HR (95% CI) derived from the Cox proportional hazards regression model analysis

*Abbreviations*: *CFA* common femoral artery, *CI* confidence interval, *HR* hazard ratio, *IVUS* intravascular ultrasound, *MLA* minimum lumen area, *PACSS* Peripheral Artery Calcium Scoring System

## Discussion

The results of our study demonstrated that the use of fracking technique in the treatment of calcified CFA lesions resulted in significantly better procedure efficacy and comparable safety outcomes compared to conventional balloon angioplasty without scaffolds. Our study also indicated that a larger postprocedural MLA, as estimated by IVUS, was independently associated with a lower incidence of restenosis. Notably, a postprocedural MLA of 16.0 mm^2^ was identified as a specific cut-off value for preventing patency loss. These findings may help guide clinical decisions and improve patient outcomes in the treatment of calcified CFA lesions.

Various treatments, such as surgical endarterectomy, scaffolds, and atherectomy devices, have been utilized to increase lumen area and reduce restenosis in CFA disease (Gallagher et al. 2011; Gouëffic et al. 2017; Brodmann et al. 2019; Kuo et al. 2019). However, balloon angioplasty has demonstrated suboptimal results with a 1-year primary patency rate of 72.4% and a bailout stenting rate of 36.9%, due to insufficient luminal gain after balloon dilatation (Bonvini et al. 2011). In a previous comparative study, directional atherectomy combined with DCB resulted in a higher 1-year primary patency rate (88%) than DCB angioplasty alone (68%) (Stavroulakis et al. 2018). These results suggest that a plaque modification strategy to maximize lumen area can improve restenosis when treating CFA lesions. Our study found that the residual stenosis, postprocedural MLA, and plaque burden results were significantly better in the fracking group than in the balloon group, which may lead to optimal outcomes similar to those achieved with surgical endarterectomy and atherectomy device use. Furthermore, the main purpose of fracking is to modify deep calcification, similar to lithoplasty (Dini et al. 2019). A retrospective study comparing lithoplasty and atherectomy with DCB for treating calcified CFA lesions demonstrated that the 18-month cumulative TLR was identical for both (79.4% and 91.2%, *P =* 0.166) (Baig et al. 2022), and the procedural complication rates were 3.0% and 5.7%, respectively. The present study showed that the incidence of procedural-related complications in the fracking group was not more than that in the balloon group. Although additional punctures were made while fracking was performed, hematoma was observed at the sheath insertion site rather than the fracking puncture sites. An 18-gauge needle was used to perform fracking in this study, and hemostasis of the puncture may be easily achieved with balloon dilation after fracking. Additionally, no distal embolization was documented during the fracking procedure. Superficial calcification is less susceptible to fracking than deep calcification and may have acted as a barrier to embolization (Haraguchi et al. 2021).

This study’s findings indicated that achieving a postprocedural MLA of more than 16.0 mm^2^ was an independent predictor of reduced restenosis risk at 1 year after treatment for calcified CFA lesions. A previous study demonstrated that calcified lesions and residual stenosis were independent predictors of restenosis (Soga et al. 2013). Severely calcified lesions, particularly those with a calcification arch over 180°, prevented lumen expansion during peripheral interventions (Fujihara et al. 2019). However, this study found that severely calcified lesions were not a relevant predictor of restenosis. Even though lesions with calcification angles exceeding 180° were more frequently observed in the fracking group than the balloon group, the fracking technique, which was utilized in addition to balloon angioplasty, effectively cracked calcification and resulted in a larger postprocedural MLA compared to conventional balloon angioplasty alone. The average MLA in the balloon group was significantly smaller than the cut-off value of 16.0 mm^2^ identified in this study, which may have contributed to the lower patency rate compared to the fracking group. Previously, no study has reported a specific cut-off value from the IVUS examination that could predict restenosis of CFA lesions. On the other hand, a study of femoropopliteal lesions demonstrated that a minimum stent area < 12.0 mm^2^ was associated with 1-year restenosis after Zilver PTX stent (Cook Co. Bloomington, IN., USA) implantation (Iida et al. 2015), and MLA < 12.7 mm^2^ after DCB angioplasty was shown to predict restenosis (Horie et al. 2022). Notably, the cut-off value in our study was larger than those reported in previous studies of femoropopliteal lesions, which may be explained by the larger diameter of CFA compared to femoropopliteal artery. This study excluded scaffolds and focused exclusively on balloon angioplasty, including DCB angioplasty. In contrast to scaffolds, balloon angioplasty cannot maintain long-term lumen area in the chronic phase. Therefore, an initial minimum lumen area (MLA) after revascularization may be an essential determinant of patency. Therefore, a modification strategy should be implemented alongside balloon angioplasty to achieve an MLA larger than 16.0 mm^2^ in calcified CFA lesions. Fracking was performed, with a mean of 2.4 ± 0.8 times (1–4 times) and an average duration of 10.1 ± 3.8 min (4–18 min). Additionally, the fracking group had a significantly shorter radiation time than the balloon group. Fracking is a minimally invasive procedure that involves using an 18-gauge needle in addition to conventional balloon angioplasty. Therefore, this technique is cost-effective and has been shown to provide significant clinical benefits for patients with calcified CFA lesions undergoing peripheral intervention.

The current study had some limitations. First, the retrospective, nonrandomized, single-center, and observational design of the study might have led to case selection bias and nonrandom assignment. The small sample size may have affected the reliability of the results. Second, the effectiveness of the fracking technique utilized in the study was dependent on the operator’s skill, which could have affected the outcomes. Third, scaffolds and atherectomy devices were not used in the study, which may limit the applicability of the results. Fourth, only calcified lesions were included, and thus, the efficacy of fracking in treating noncalcified lesions remains unknown. Finally, since the follow-up period was limited to 1 year, the clinical outcomes in the chronic phase are still unknown. Therefore, further studies with larger sample sizes, longer follow-up periods, and randomized designs are needed to evaluate the long-term safety and efficacy of this treatment approach.

## Conclusions

This study demonstrated that the utilization of fracking with IVUS guidance resulted in lower rates of 1-year restenosis and reintervention compared to conventional balloon angioplasty for the treatment of calcified CFA lesions. The incidence rates of procedural complications, major amputation, and all-cause death were not significantly different between the two procedures. A postprocedural MLA of less than 16.0 mm^2^ was associated with an increased risk of restenosis. Therefore, a calcified plaque modification strategy is recommended in addition to balloon angioplasty if the postprocedural MLA is less than 16.0 mm^2^ following conventional endovascular procedures.

## Data Availability

The datasets used and analyzed during the current study are available from the corresponding author upon reasonable request.

## Abbreviations

ABI: Ankle-brachial index
CFA: Common femoral artery
DCB: Drug-coated balloon
IVUS: Intravascular ultrasound
MLA: Minimum lumen area
PACSS: Peripheral arterial calcium-scoring system
ROC: Receiver operating characteristics
TLR: Target lesion revascularization
